# Supplementary material for: The influence of the age-period-cohort effects on the temporal trend mortality from schistosomiasis in Brazil from 1980 to 2014
Source: PLoS One. 2020 Apr 23;15(4):e0231874. doi: 10.1371/journal.pone.0231874 (PMC7179843; doi:10.1371/journal.pone.0231874)
Supplement: Attachment — Submitted filename: Response to Reviwers.docx [file pone.0231874.s001.docx]

**Responses to the reviewers' comments and descriptions of the changes we have made in the manuscript:**

**The authors of the manuscript “The influence of the age-period-cohort effects on the temporal trend mortality from schistosomiasis in Brazil from 1980 to 2014” (#PONE-D-19-22417) thank the editor and reviewers of PLoS ONE for their insightful comments and recommendations that helped to improve our study. We have appreciated all the considerations. We have also provided a new revised version of the manuscript that will hopefully cover all the points raised by the reviewers. Below, detailed answers are provided to specific concerns raised by each note of each referee. The answers are in bold.**

Dear PhD Simões,

Thank you for submitting your manuscript to PLOS ONE. After careful consideration, we feel that it has merit but does not fully meet PLOS ONE’s publication criteria as it currently stands. Therefore, we invite you to submit a revised version of the manuscript
that addresses the specific points raised during the review process.

We would appreciate receiving your revised manuscript by Mar 13 2020 11:59PM. When you are ready to submit your revision, log on to https://www.editorialmanager.com/pone/ and select the 'Submissions Needing Revision' folder to locate your manuscript file.

If you would like to make changes to your financial disclosure, please include your updated statement in your cover letter.

To enhance the reproducibility of your results, we recommend that if applicable you deposit your laboratory protocols in protocols.io, where a protocol can be assigned its own identifier (DOI) such that it can be cited independently in the future. For instructions see: <http://journals.plos.org/plosone/s/submission-guidelines#loc-laboratory-protocols>

Please include the following items when submitting your revised manuscript:

- A rebuttal letter that responds to each point raised by the academic editor and reviewer(s). This letter should be uploaded as separate file and labeled 'Response to Reviewers'.
- A marked-up copy of your manuscript that highlights changes made to the original version. This file should be uploaded as separate file and labeled 'Revised Manuscript with Track Changes'.
- An unmarked version of your revised paper without tracked changes. This file should be uploaded as separate file and labeled 'Manuscript'.

Please note while forming your response, if your article is accepted, you may have the opportunity to make the peer review history publicly available. The record will include editor decision letters (with reviews) and your responses to reviewer comments. If eligible, we will contact you to opt in or out.

We look forward to receiving your revised manuscript.

Kind regards,

David Joseph Diemert, M.D.

Academic Editor

PLOS ONE

Journal Requirements:

When submitting your revision, we need you to address these additional requirements:

1. Please ensure that your manuscript meets PLOS ONE's style requirements, including those for file naming. The PLOS ONE style templates can be found at http://www.plosone.org/attachments/PLOSOne_formatting_sample_main_body.pdf and http://www.plosone.org/attachments/PLOSOne_formatting_sample_title_authors_affiliations.pdf

2. Please amend the subsection category “[FOR JOURNAL STAFF USE ONLY]” for your manuscript. Unfortunately, this is not a valid category. At this time, please choose one or more subsections that best represent the topic(s) of your study.

3. We note that you have stated that you will provide repository information for your data at acceptance. Should your manuscript be accepted for publication, we will hold it until you provide the relevant accession numbers or DOIs necessary to access your data. If you wish to make changes to your Data Availability statement, please describe these changes in your cover letter and we will update your Data Availability statement to reflect the information you provide.

4. Please include your tables as part of your main manuscript and remove the individual files. Please note that supplementary tables (should remain/ be uploaded) as separate "supporting information" files

[Note: HTML markup is below. Please do not edit.]

Reviewers' comments:

Reviewer's Responses to Questions

**Comments to the Author**

1. Is the manuscript technically sound, and do the data support the conclusions?

The manuscript must describe a technically sound piece of scientific research with data that supports the conclusions. Experiments must have been conducted rigorously, with appropriate controls, replication, and sample sizes. The conclusions must be drawn appropriately based on the data presented.

Reviewer #1: Yes

Reviewer #2: No

2. Has the statistical analysis been performed appropriately and rigorously?

Reviewer #1: I Don't Know

Reviewer #2: Yes

3. Have the authors made all data underlying the findings in their manuscript fully available?
The [PLOS Data policy](http://www.plosone.org/static/policies.action#sharing) requires authors to make all data underlying the findings described in their manuscript fully available without restriction, with rare exception (please refer to the Data Availability Statement in the manuscript PDF file). The data should be provided as part of the manuscript or its supporting information, or deposited to a public repository. For example, in addition to summary statistics, the data points behind means, medians and variance measures should be available. If there are restrictions on publicly sharing data—e.g. participant privacy or use of data from a third party—those must be specified.

Reviewer #1: Yes

Reviewer #2: Yes

4. Is the manuscript presented in an intelligible fashion and written in standard English?

PLOS ONE does not copyedit accepted manuscripts, so the language in submitted articles must be clear, correct, and unambiguous. Any typographical or grammatical errors should be corrected at revision, so please note any specific errors here.

Reviewer #1: No

Reviewer #2: No

5. Review Comments to the Author

Please use the space provided to explain your answers to the questions above. You may also include additional comments for the author, including concerns about dual publication, research ethics, or publication ethics. (Please upload your review as an attachment if it exceeds 20,000 characters)

Reviewer #1: The theme of the manuscript is important regarding mortality and population infected with Schistosoma mansoni in Brazil. It also compares different age groups, as well as different populations from the socioeconomic point of view. However the English is poor and must be improved.

**Answer:**

**We are grateful for the reviewer's contribution and we certify that we have reviewed the entire writing of the manuscript, as well as the adequacy of the language.**

Reviewer #2: This study examined the effects of age, period and birth cohort (APC Effects) on the temporal evolution of schistosomiasis mortality in Brazil, according to sex and the five Brazilian geographic regions from 1980 to 2014. The results show that about 20,000 schistosomiasis deaths were recorded in Brazil during the study period. The highest mortality rates from schistosomiasis occurred at the most advanced ages, in men and in the Northeast region. The mortality rates increased with increasing age, declined over the years, and were higher for older generations or birth cohorts.
The study has great relevance in the area. However, there are important issues/points that need to be improved in the manuscript before being reconsidered:

-I suggest a comprehensive review of the English written in the manuscript (typographical / grammatical errors and scientific writing);

**Answer:**

**We are grateful for the reviewer's contribution and we certify that we have reviewed the entire writing of the manuscript, as well as the adequacy of the language.**

Title:

-Lines 1-2: Please identify the time period (1980-2014) and replace “cohort effects” by “age-period-cohort effects”;

**Answer:**

**We changed the title to:** “**The influence of the age-period-cohort effects on the temporal trend of mortality from schistosomiasis in Brazil from 1980 to 2014**”.

Abstract:

-Background: Please include the overall objective of the study;

**Answer:**

**We added this sentence to the summary “In this study, the temporal change in mortality was evaluated in relation to the effects of age, period and birth cohort, in Brazil and regions, from 1980 to 2014”.**

-Methods: Please identify the study design; Are the population data extracted from the SIM or IBGE?

**Answer:**

**We completed the first sentence of the methods section: “This is a population-based longitudinal ecological study to analyze the time trend of schistosomiasis mortality rates in Brazil” (page 6, line 137). In addition, we clarify where population data were obtained: “Population data were** **obtained from the IBGE website (Brazilian Institute of Geography and Statistics) [16]” (page 7, line 164).**

-Results: The description of the findings is confusing and difficult to understand; -Please replace "Midwest" with "Central-West" in the entire manuscript (e.g., Line 40);

**Answer:**

**We reviewed and rewrote part of the results section. In addition, we made the suggested replacement for "Central-West".**

-The conclusions should be focused/based on the key findings of the study.
**Answer:**

**We reviewed and rewrote the conclusions:**

**“The birth cohorts had a great influence on the decreasing trend of schistosomiasis mortality in Brazil. This result may be due to the interaction between demographic changes and greater access to health and sanitation services, in addition to the impact of schistosomiasis control measures experienced by younger cohorts.”**

Author summary:

-Most of this section is based on the description of the background, with an insufficient description of the main findings of the study.

**Answer:**

**We rewrote this section with a focus on the methodology used and the results obtained:**

**“Schistosomiasis infection is prevalent in tropical and subtropical areas in poor communities, without access to drinking water and without adequate sanitation, affecting millions of people worldwide. In Brazil, *Schistosomiasis mansoni* is endemic in a large part of the territory and considered an important public health problem. In this study, we analyzed the influence of age, period and birth cohort (APC effects) on the temporal evolution of schistosomiasis mortality in Brazil and its geographic regions, between 1980 and 2014. The APC effects varied according to sex and regions. Men were at risk of dying between 25 and 54 years of age, while risk for women occurred after seventy years of age. Birth Cohorts had the greatest influence on the trend of decreasing mortality, with a protective effect on death among people born after 1973. Demographic changes and greater access to health experienced by younger generations may have a role important in decreasing deaths from the disease.”**

Introduction:

-This section is relatively long and could be shortened focusing on contextualization and justification of the study problem.

**Answer:**

**We have reviewed and summarized this section, focusing on the problems highlighted by the reviewer.**

Methods:

-Lines 146-156: Please include a more updated estimate of the Brazilian population (e.g., 210.1 million in 2019);

**Answer:**

**We updated population estimates for 2019:**

**“****This is a population-based longitudinal ecological study to analyze the time trend of schistosomiasis mortality rates in Brazil. The country is divided into 26 states and a federal government grouped into five major regions (North, Northeast, Southeast, South and Central-West) with different geographical, economic and cultural characteristics. The Southeast (88,072,407 inhabitants) is the most populous and economically most developed region, and the South region (30,036,030 inhabitants) has the best human development indexes. On the other hand, the Central-West (16,293,774 inhabitants) has an economy based on agriculture and livestock, the Northeast (57,883,049 inhabitants) has the lowest human development rates and the North (18,373,753 inhabitants), which includes the Amazon rainforest, has low population density and ranks second with the lowest human development rates.** **Population data were obtained from the IBGE website (Brazilian Institute of Geography and Statistics).”**

-Lines 157-181: Please include more information and details of the national databases used (e.g. SIM database, death certificate [DO] as standard death registration document, etc.) and explain and clarify how mortality and population data were extracted from national databases (downloading the SIM microdata or data tabulation through the DATASUS website); In addition, please specify the period/year of data collection and clarify whether schistosomiasis deaths were extracted as a underlying or multiple causes of death (underlying and associated causes of death).

**Answer:**

**We rewrote the sentence related to obtaining the data we used, making it clearer and more detailed. In addition, we added the collection period and the type of death analyzed (underlying death).**

**Page 7, lines 149-162: “The annual death records between 1980 and, specific for age, were extracted from the Mortality Information System of the SUS (Unified Health System) Department of Information Technology (SIM-DATASUS) of the Ministry of Health of Brazil (DATASUS, 2017). The categories of underlying deaths** **selected in the SIM were Schistosomiasis - codes 120 (ICD-9 until 1995) and B65 (ICD-10 after 1996). SIM was created for the regular collection of data on mortality in the country, allowing the collection of data in a comprehensive way, in order to subsidize the different spheres of management in public health. Death certificates contain demographic (age, gender, education, race, marital status, municipality of residence and municipality of occurrence of death) and clinical information (underlying and associated causes of death). It is the physicians’ responsibility to complete the death certificates. Until 1995, reference codes were based on the International Classification of Diseases (ICD) in its 9th revision, and after 1996, they were based on the 10th revision of the ICD. All data were collected in November 2017 [13].”**

**Page 7, lines 147-48: “****Population data were obtained from the IBGE website (Brazilian Institute of Geography and Statistics).**

Results:

-Please replace "basic cause" with "underlying cause" in the entire manuscript (e.g., Line 229);
**Answer:**

**We made the suggested replacement.**

-Please stratify the description of the findings in more subsections.

**Answer:**

**We stratified the description of the findings in the subsections: "Describing the data", "****APC effects on mortality in Brazil" and "APC effects on mortality in regions".**

-Line 233: Is the value of this mortality rate correct (2.99 deaths/100,000 inhabitants)?

**Answer:**

**The value is correct, but it is the crude rate calculated for the entire period of analysis. We rewrote the sentence.**

**Page 10, lines 233-35: “The crude death rate from schistosomiasis was 2.99 deaths per 100,000 inhabitants during the entire period analyzed (1980-2014). The standardized annual average rate was 0.45 deaths per 100,000 inhabitants.”**

-Discussion:

-The discussion is long and somewhat repetitive in some parts, requiring a comprehensive review. I recommend that the authors show each important point found in the study in a logical sequence according to the Results section, immediately before discussing possible reasons found in the literature. It would be clearer if each paragraph represents a single idea. In addition, please do not repeat the findings described in the Results section;

**Answer:**

**We have reviewed and summarized this section, focusing on the issues highlighted by the reviewer.**

-Limitations: Please include the limitations on the quality and coverage of mortality data in Brazil over the study period;

**Answer:**

**We included a sentence about the quality and coverage of the data as limitations of the study.**

**Page 15, lines 411-18: “As limitations of this study, it can be mentioned that the number of deaths due to schistosomiasis is difficult to estimate due to hidden pathologies, such as liver and kidney failure, bladder cancer and ectopic pregnancy due to female genital schistosomiasis. Another counterpoint of this work is the quality of information and the coverage of the mortality information system. Although we performed the correction of death records for ill-defined causes, in addition to ignored sex and age, we did not correct the underreporting of deaths and, therefore, schistosomiasis mortality rates may be underestimated [7,9].”**

-Please finalize the Discussion section with the conclusion(s) of the study.

**Answer:**

**We conclude the Discussion section with a paragraph of conclusions.**

**Page 15, lines 419-24: “This study showed the important role of younger birth cohorts in the overall reduction of schistosomiasis mortality over time in Brazil and geographic regions. This effect reflects the effectiveness of disease control programs and improvements in access to health and socioeconomic conditions of the population over time. The effects of age, period and cohort also varied according to sex and geographic regions, corroborating the different habits and living conditions of the Brazilian population.”**

6. PLOS authors have the option to publish the peer review history of their article ([what does this mean?](https://journals.plos.org/plosone/s/editorial-and-peer-review-process#loc-peer-review-history)). If published, this will include your full peer review and any attached files.

If you choose “no”, your identity will remain anonymous but your review may still be made public.

**Do you want your identity to be public for this peer review?** For information about this choice, including consent withdrawal, please see our [Privacy Policy](https://www.plos.org/privacy-policy).

Reviewer #1: No

Reviewer #2: No

[NOTE: If reviewer comments were submitted as an attachment file, they will be attached to this email and accessible via the submission site. Please log into your account, locate the manuscript record, and check for the action link "View Attachments". If this link does not appear, there are no attachment files to be viewed.]

While revising your submission, please upload your figure files to the Preflight Analysis and Conversion Engine (PACE) digital diagnostic tool, <https://pacev2.apexcovantage.com/>. PACE helps ensure that figures meet PLOS requirements. To use PACE, you must first register as a user. Registration is free. Then, login and navigate to the UPLOAD tab, where you will find detailed instructions on how to use the tool. If you encounter any issues or have any questions when using PACE, please email us at [figures@plos.org](mailto:figures@plos.org). Please note that Supporting Information files do not need this step.
